# Investigating the Obesity Paradox in Colorectal Cancer: An Analysis of Prospectively Collected Data in a Diverse Cohort

**DOI:** 10.3390/cancers16172950

**Published:** 2024-08-24

**Authors:** Shria Kumar, Catherine Blandon, Alla Sikorskii, David E. Kaplan, Shivan J. Mehta, Grace L. Su, David S. Goldberg, Tracy E. Crane

**Affiliations:** 1 Division of Digestive Health and Liver Diseases, Department of Medicine, Miller School of Medicine at the University of Miami, Miami, FL 33136, USA; 2 Sylvester Comprehensive Cancer Center, Miller School of Medicine at the University of Miami, Miami, FL 33136, USA; tecrane@med.miami.edu; 3 Department of Psychiatry, Michigan State University, East Lansing, MI 48824, USA; 4 Division of Gastroenterology and Hepatology, University of Pennsylvania, Philadelphia, PA 19104, USA; 5 Gastroenterology Section, Corporal Michael J. Crescenz VA Medical Center, Philadelphia, PA 19104, USA; 6 Division of Gastroenterology, University of Michigan, Ann Arbor, MI 48109, USA; 7 Medicine Service, Lieutenant Colonel Charles S. Kettles VA Medical Center, Ann Arbor, MI 48105, USA; 8 Division of Medical Oncology, Department of Medicine, Miller School of Medicine at the University of Miami, Miami, FL 33136, USA

**Keywords:** colorectal cancer, obesity paradox, group-based trajectory modeling, disparities

## Abstract

**Simple Summary:**

Obesity is a clear risk factor for future colorectal cancer (CRC), but its impact on mortality remains to be fully elucidated. Prior studies have had methodologic limitations, and key variables remain under-investigated. In this study of prospectively collected data from the Multiethnic Cohort, pre-diagnosis BMI trajectories were not associated with CRC-specific or all-cause mortality. Factors associated with mortality included African American race or Hawaiian ethnicity, smoking, and diabetes. Accounting for long-term BMI trajectories before diagnosis and other relevant factors demonstrates that obesity is not protective of mortality after CRC diagnosis.

**Abstract:**

Background: Prior studies are inconclusive regarding the effect of obesity on mortality in persons with colorectal cancer (CRC). We sought to determine the association of pre-diagnosis body mass index (BMI) trajectories on mortality after CRC diagnosis. Methods: Utilizing the Multiethnic Cohort, we included adults aged 18–75 between 1 January 1993 and 1 January 2019 with a diagnosis of CRC and at least three available BMIs. The primary exposure, BMI, was subjected to group-based trajectory modeling (GBTM). We evaluated all-cause and CRC-specific mortality, using Cox proportional hazard (PH) models. Results: Of 924 persons, the median age was 60 years, and 54% were female. There was no statistically significant association between pre-cancer BMI trajectory and either all-cause or cancer-specific mortality. In competing risk analysis, the risk of CRC-specific mortality was higher for African Americans (HR = 1.56, 95% CI [1.00–2.43], *p* = 0.048) and smokers (HR = 1.59, 95% CI [1.10–2.32], *p* = 0.015). Risk of all-cause mortality was higher for Hawaiian persons (HR = 2.85, 95% CI [1.31–6.21], *p* = 0.009) and persons with diabetes (HR = 1.83, 95% CI [1.08–3.10], *p* = 0.026). Conclusions: Pre-diagnosis BMI trajectories were not associated with mortality after CRC diagnosis, whereas race/ethnicity, diabetes, and smoking were associated with an increased risk of death. Our findings suggest the obesity paradox alone does not account for mortality after CRC diagnosis.

## 1. Introduction

There is substantial evidence that obesity is a risk factor for future colorectal cancer (CRC), but its impact on mortality remains to be fully elucidated [1,2,3,4]. Obesity is a chronic disease associated with significant morbidity and mortality, so it would follow that obesity would be associated with worse CRC outcomes [5,6,7,8,9]. Yet there is marked ambiguity in this hypothesis, underlined by the term “obesity paradox”, in which obesity appears to confer protection against cancer-related mortality. There is no clear consensus, with some studies showing a possible protective effect of obesity on mortality while others do not [10,11,12]. For example, a 2016 investigation of persons diagnosed with CRC who underwent surgery found that the lowest risk of mortality was in those persons with a body mass index (BMI) of 28, indicating obesity [11]. A 2022 meta-analysis of 16 studies also found that CRC patients with a higher BMI appear to have reduced mortality compared with normal-weight CRC patients [1]. On the other hand, a 2021 meta-analysis found that obesity was associated with increased mortality in CRC patients [1]. Since there are recommendations for both diet and exercise for cancer survivors, a clearer understanding of the role of obesity in cancer mortality is needed, particularly to avoid confusing messages to patients and to further refine the national recommendations and guidelines for cancer survivors.

There are plausible reasons why obesity may improve survival, including survival advantage because of greater physiologic resilience and metabolic reserves and differential tumor characteristics [13,14]. That is, persons with obesity may be able to better tolerate cancer cachexia, as well as weight loss associated with cancer treatments. However, obesity is a broad classifier, not always indicative of overall health and with varying degrees of severity. Moreover, given the nature of exploring the obesity paradox in large datasets, where weight is usually examined at or after a cancer diagnosis, studies exploring the association can be subject to methodological flaws, including confounding, reverse causality, and effect modification between exposure and outcome [7,8]. Prior studies have been heterogenous, evaluating BMI at varying time points (pre-, peri-, or post-diagnosis) and lacking data on long-term pre-diagnosis trajectories [15,16]. In addition, even though it is well-known that CRC and obesity rates and outcomes differ across racial and ethnic groups [17,18,19], prior studies have not been conducted in cohorts with sufficient diversity and have not uniformly adjusted for stage. As a result, much has been written about the obesity paradox, but key variables remain under investigated. To address this gap and evaluate the obesity paradox, we used long-term pre-diagnosis BMI data from a large longitudinal prospective diverse cohort and related BMI trajectories to mortality.

## 2. Materials and Methods

In this analysis of existing data from the prospective Multiethnic Cohort study (MEC), we identified persons with CRC, their weight (BMI) trajectories pre-diagnosis, and performed multivariable Cox PH regression analyses to determine the association between weight trajectory pre-diagnosis and mortality. This study was approved by the University of Miami Institutional Review Board.

### 2.1. Study Population

The MEC is a National Cancer Institute (NCI)-funded prospective cohort study established in 1993 [20], with >215,000 individuals living in Hawaii and California of five main ethnicities: Japanese-, African-, and white-Americans, Native Hawaiians, and Hispanic/Latinos. The MEC follows individuals over time to collect data and inform the association of individual and lifestyle factors with chronic diseases, including cancer [20]. The MEC has also been the basis for robust cancer epidemiology studies [21,22,23,24,25,26]. The MEC ascertains cancer via linkage to the NCI SEER Program, a high-quality and high-fidelity cancer registry [27].

We included adults aged 45–75 between 1 January 1993 and 1 January 2019 with a CRC diagnosis and at least three pre-diagnosis BMIs to ensure adequate engagement and follow-up. The BMI was self-reported from four separate questionnaires, which were separated by at least 1 year. We excluded people with cancer (except non-melanoma skin cancers) prior to inclusion into the cohort or those that developed other cancers during follow-up, as this may have impacted BMI.

### 2.2. Outcome and Exposures

The primary outcome was time to (a) all-cause and (b) CRC-specific mortality. The primary exposure was BMI (and not weight). We did not include any BMI within 1 year prior to death [16,28]. Longitudinal BMI data were subjected to group-based trajectory modeling (GBTM), a type of trajectory analysis in which discrete patterns of changes over time were identified, and individuals were assigned to a category based on maximum likelihood estimation [29,30,31,32,33,34]. This relatively novel methodology helped organize heterogeneous longitudinal data into patterns that provide more information about BMI than cross-sectional data and more nuance than cumulative exposure alone [35,36]. We identified BMI trajectories (groups of people with similar longitudinal BMI) controlling for age at the first of four assessments and accounting for slight variations in time between assessments within GBTM. In addition to age, covariates included sex, ethnicity, and known factors associated with CRC, including the following: diabetes [37], smoking [1,38], and family history [1,39]. Ethnicity is self-reported ethnicity, categorized as included on the MEC questionnaire.

### 2.3. Statistical Analysis

We summarized the distribution of patient characteristics by count (%) and median (interquartile range) and compared those that did and did not develop the outcome of interest using Fisher’s test and Wilcoxon’s signed-rank test for categorical variables and continuous variables, respectively.

In the GBTM, the number of groups was chosen based on Bayesian information criteria [40,41]. To ensure that each group had an adequate number of persons, we required that each class include at least 5% of the cohort [42,43,44,45]. We further ensured goodness-of-fit by ensuring a mean posterior probability of each class >75%, and we compared model-fitted values to observed values to ensure that the assumption of GBTM is fulfilled: that repeated measures are conditionally independent given group membership [40,42,43,44,45]. Finally, we created propensity scores for BMI trajectories to ensure sufficient overlap. 

We then conducted multivariable analysis with Cox proportional hazard (PH) regression (time to CRC-specific death), using the BMI trajectory group as the key explanatory variable. The Cox PH model was developed using separate baseline hazard functions for each CRC stage, given the differential risk of death by stage. The beta coefficients were then optimized to be applied consistently across all stages. This allowed us to stratify by stage but present one unified model. We further evaluated the potential interaction of ethnicity or sex between BMI trajectory group membership and outcome of interest in these stratified analyses. Adjusted models included prespecified variables (i.e., sex, ethnicity, CRC stage, smoking at cohort entry) and clinically significant variables (i.e., diabetes) in the model. To account for the nonlinear effects of age, age was modeled via cubic splines. A sensitivity analysis was conducted to evaluate competing risks.

We performed multiple imputations via chained equations (MICE) for 10 iterations across missing values for covariates. The statistical analysis was conducted with R version 4.3.1. Reporting of this study followed the Strengthening the Reporting of Observational Studies in Epidemiology (STROBE) reporting guidelines for cohort studies.

A priori sample size calculations were performed. For GBTM, models obtain accurate estimates of slope at a sample size of 500, which our pooled cohort satisfied [46]. We estimated a 64% survival after CRC diagnosis, and the preliminary sample size showed at least 1455 persons in the MEC [47]. We expected >99% power at a 0.05 significance level to detect a hazard ratio of 0.2 [48].

## 3. Results

We identified 924 persons with CRC who met the inclusion criteria—the median age was 60 years, and 496 (53.7%) were female (Supplemental Figure S1). Mortality was observed in 394 (42.6%) persons with a median of 1 year (IQR 0–3) after CRC diagnosis, of which 271 (68.8%) were CRC-specific. 

We first compared persons who did and did not develop the outcome of interest—mortality. Compared to those who were alive at the end of the follow-up, those who died were older at cancer diagnosis (81.5 vs. 75.0, *p* < 0.001), diabetic (11.4% vs. 7.2%, *p* = 0.03), more likely to have distant stage disease (27.4% vs. 4.3%, *p* < 0.001), and less likely to be white (19.3% vs. 27.5%, *p* = 0.02) (Table 1). There was no significant difference in median BMI at cohort entry, but those who died were more likely to have lost weight over time (% change in BMI from questionnaire first to last: −2.7 vs. 0, *p* < 0.001). Supplemental Table S1 provides the corresponding results for CRC-specific vs. other mortality. 

**Figure 1:**
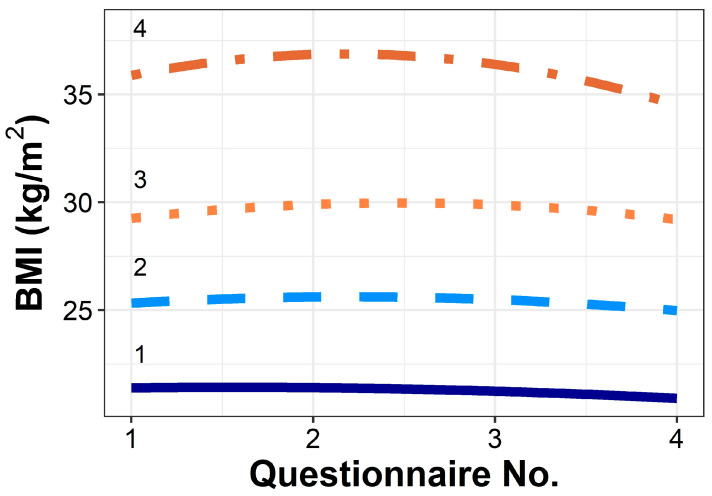
BMI trajectories of each group created using GBTM. Within the multivariable models, groups were further adjusted by age.

We then created the groups by using BMI trajectories. These groups are presented in Figure 1. Persons in Group 1 had the lowest starting BMI of 21.3 and lost 3.0% BMI over the follow-up period. Group 2 began at 25.2 and lost 2.3% BMI over the follow-up period. Group 3 began at 28.6 and lost 3.2% BMI, while Group 4 began at the highest BMI, 34.6, and lost 2.9% BMI over the follow-up period. Supplemental Table S2 describes the groups: the majority of Group 1 (91.4%) was classified as normal weight, with a median BMI of 21.3 at cohort entry. In Group 2, 91 persons (59.9%) were classified as overweight, while 61 (40.1%) were normal weight at cohort entry. Group 3 had 66 (76.7%) classified as overweight, while 17 (19.8%) were Class 1 obese. Finally, Group 4 had 29 (46.0%) classified as Class 1 obese, 19 (30.2%), Class 2 obese, and 10 (15.9%) as Class 3 obese. BMI trajectory propensity scores were created and showed sufficient overlap. 

We then conducted multivariable analyses, to investigate the association of mortality with BMI trajectories. The results of the multivariable analysis of Cox PH, showing adjusted HRs of all-cause and cancer-specific mortality, are shown in Table 2. There were no statistically significant differences among BMI trajectories in either all-cause or cancer-specific mortality; however, Group 3 (BMI 28.6 at first questionnaire) had the lowest hazard of death for all-cause mortality (HR = 0.81, 95% CI [0.61–1.08], *p* = 0.147) and cancer-specific mortality (HR = 0.80, 95% CI [0.56–1.13], *p* = 0.200), compared to Group 2 (BMI 25.2 at first questionnaire). 

**Figure 2:**
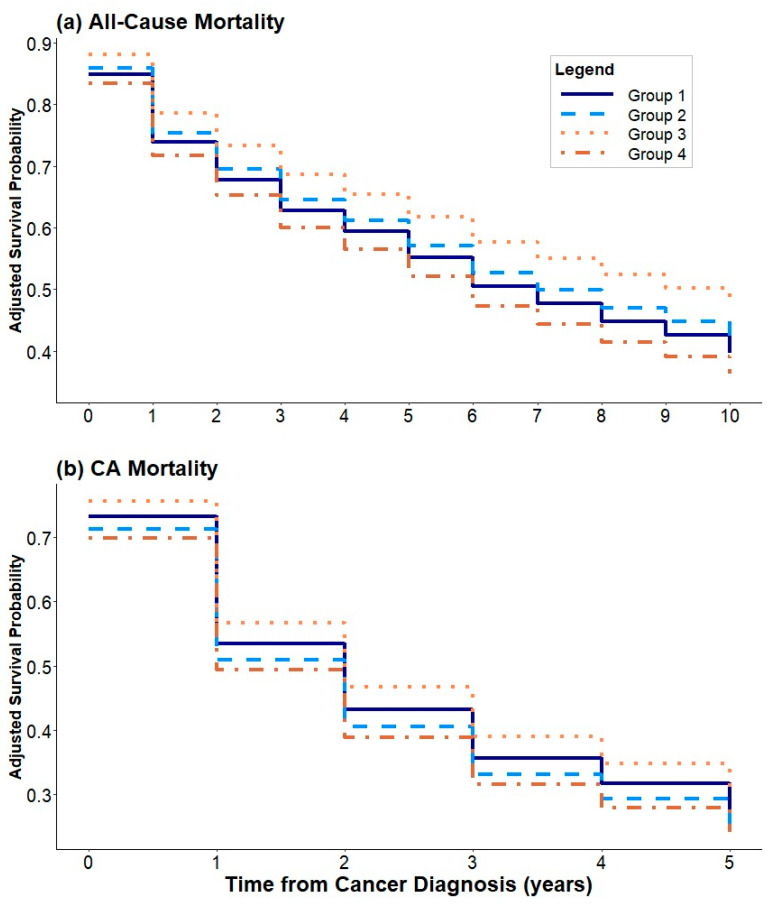
Survival curves showing (**a**) all-cause mortality and (**b**) cancer-specific mortality.

Certain ethnic groups showed a differential risk of mortality when compared to whites. For all-cause mortality, African Americans had a 58% increased risk of death (HR = 1.58, 95% CI [1.09–2.29], *p* = 0.016). Hawaiian and Japanese persons had 52% (HR 0.48, 95% CI [0.27–0.87], *p* = 0.015) and 32% (HR = 0.68, 95% CI [0.48–0.96], *p* = 0.029) lower risk of cancer-specific mortality, respectively. Current smokers at cohort entry and persons with a history of smoking had 66% (HR = 1.66, 95% CI [1.21–2.29], *p* = 0.002) and 28% (HR = 1.28, 95% CI [1.01–1.63], *p* = 0.044) increased risk of all-cause mortality, respectively. There was a nonlinear association between the age of CRC diagnosis and all-cause mortality with increasing risk from ages 65 to 88 (Supplemental Figure S2). Figure 2 depicts the survival curves for both all-cause and cancer-specific mortality, again showing no statistically significant differences among BMI trajectories in either all-cause or cancer-specific mortality.

A sensitivity analysis was then conducted, evaluating competing risks. The primary outcome was CRC-specific mortality, with all-cause mortality considered a competing risk. The results are presented in Table 3. There was still no statistically significant association between BMI trajectory and mortality. The risk of CRC-specific mortality was significantly higher for African Americans (HR 1.56, 95% CI [1.00–2.43], *p* = 0.048) and for smokers (HR 1.59, 95% CI [1.10–2.32], *p* = 0.015). The competing risk of all-cause mortality was higher for Hawaiian persons (HR 2.85, 95% CI [1.31–6.21], *p* = 0.009) and persons with diabetes (HR 1.83, 95% CI [1.08–3.10], *p* = 0.026).

## 4. Discussion

In this study evaluating the potential impact of pre-diagnosis BMI trajectories on mortality after CRC diagnosis, we found no statistically significant effect of BMI trajectory on either all-cause or cancer-specific mortality in multivariable models. Our study includes long-term, prospectively collected data, and improves upon prior studies by using advanced methodologies and considering relevant confounders. Our findings suggest that the obesity paradox does not hold true, after accounting for pre-diagnosis BMI trajectories (starting approximately two decades prior to CRC diagnosis) and accounting for important factors, such as ethnicity and comorbidities. 

Many studies evaluating the obesity paradox rely on at-diagnosis BMI, and therefore a plausible hypothesis for why obesity seems protective against mortality in these studies includes greater body robustness or excess caloric reserve [12,49]. However, these findings do not equate to a protective effect of pre-diagnostic obesity. In fact, severe obesity has almost always been associated with an increased risk of death; in this study, our point estimates suggest the same [50]. The main outstanding question in the obesity paradox is whether being overweight or only slightly obese relative to ideal body weight is protective. We found only non-significant trends toward decreased risk of death among persons who were obese many years prior to CRC diagnosis. Other studies (including those that also used pre-diagnosis BMI) have even found that pre-diagnosis obesity is associated with worse survival. A 2012 study of the Cancer Prevention Study-II Nutrition Cohort found that compared to normal weight, obesity (7 years pre-diagnosis) was associated with higher all-cause mortality [51]. Another 2014 study of the European Prospective Investigation into Cancer and Nutrition found similar results [52]. Both of these studies predominantly focused on white populations. Our inclusion of diverse populations enhances prior studies and ensures the generalizability of our findings to the US population [15]. 

The inclusion of relevant covariates, including ethnicity, is of paramount importance, and our study findings depict the complexity of interrogating the obesity paradox. Our finding that smoking and diabetes are associated with mortality are well-known and add to the abundant literature demonstrating that a healthy lifestyle should be adopted [53]. The findings of ethnicity are more complex. While initial models showed that African Americans and smokers had an increased risk of all-cause mortality, and Hawaiian and Japanese persons had a lower risk of CRC-specific mortality, a competing risk analysis demonstrated increased CRC-specific mortality among African Americans and smokers, and an increased risk of all-cause mortality among Hawaiian persons and those with diabetes. The previously noted lower risks of CRC-specific mortality in persons of Hawaiian and Japanese ethnicity were no longer noted. Disparities among African Americans regarding all-cause mortality are well-known [54]. Similarly, the elevated risk of CRC-specific mortality for African Americans is well-documented, and it has been noted that African Americans have 20% higher CRC incidence and 40% higher CRC-specific mortality [17,18]. The increased mortality is likely multi-factorial, but there are structural factors at play [55]. For example, African Americans are less likely to receive treatment after CRC diagnosis but have higher cancer-attributable costs [56]. It is thought that some of these disparities are attributable to insurance coverage, but there are also likely discrepancies in the receipt of guideline-concordant care that underlines the importance of policy-driven changes to mitigate disparities [57,58].

Our findings that persons of Hawaiian ethnicity have increased all-cause mortality are mirrored by prior literature. A 2022 study using the National Cancer Database (NCDB) established that native Hawaiians experience health disparities in cancer outcomes, and comorbidity burden may explain some of this risk [59]. The NCDB is hospital-based (not population-based), and lacks information including cause of death, which our study is able to identify. Asians are a heterogeneous group—that persons of Hawaiian ethnicity have a differential risk of death compared to persons of Japanese ethnicity shows that disaggregating Asian populations in epidemiologic studies is critical. The MEC is unique in its composition, and as future cohort studies are built, efforts should be made to disaggregate Asian Americans to ensure populations that experience disparities are not hidden [60].

Establishing whether the obesity paradox stands is of paramount importance. At the same time, the difficulty in studying the obesity paradox in CRC is well-established. There are multiple methodologic issues and caveats, borne from the limitations of available data, which are described herein. On the other hand, there are plausible reasons why the obesity paradox could exist. It is possible that obesity-related CRCs have distinct biological mechanisms or that persons with obesity have excess energy reserves, capable of withstanding the impact of tumors and tumor-directed treatments. Disentangling the obesity paradox is important to minimize confusing messaging to persons with cancer. 

This study overcomes the limitations of prior studies, as we account for relevant factors (including smoking, sex, and stage), minimize findings attributable to reverse causation by using repeated measurements, decrease collider bias by accounting for smoking, and evaluate a diverse cohort of individuals. Finally, we also explore competing risks from all-cause mortality. However, our study has limitations, including that intentionality in weight changes is difficult to ascertain, we rely on self-reported BMI, and BMI remains an imperfect measure, particularly in Asian Americans [61,62,63,64,65]. Body composition may be more informative than BMI in explaining mortality, and this is an ongoing area of study [66]. There is selection bias in who is enrolled and engaged with the MEC, which may limit broad generalizability. There are also limitations in data collection, as we do not have detailed access to CRC screening utilization and outcomes or medical conditions and symptomology. There may also be other unmeasured confounders such as genetic predisposition to CRC or bariatric surgery, as well as diet (including red and processed meats) and alcohol intake, a limitation of all large cohort studies. While we demonstrate disparities, we are unable to account for potential reasons (access to care, receipt of guideline-concordant care) to explain these. Our mortality was higher than expected [67]; however, as we are looking at BMI many years prior to CRC diagnosis, our evaluation of the obesity paradox remains valid. Finally, despite a priori sample size calculations, our study was underpowered, which may have led to an inability to detect differences where they exist, though most of the point estimates are similar to prior literature. 

## 5. Conclusions

In a comprehensive assessment that evaluates pre-diagnosis BMI trajectories beginning up to two decades prior to CRC diagnosis, we do not find a protective effect of obesity on mortality after CRC diagnosis, and in fact, find that pre-diagnosis BMI is not statistically associated with mortality. We do find that African American race and Hawaiian ethnicity, diabetes, and smoking are associated with an increased risk of death. While healthy lifestyles should continue to be promoted (avoidance of obesity, smoking, and diabetes), continued efforts should be made to reduce disparities and decrease cancer deaths among racial and ethnic minorities. 

## Figures and Tables

**Table 1:** Cohort characteristics.

Variable	All-Cause Mortality N = 394	No Mortality N = 530	*p*-Value
Age (years), Median (Q1, Q3)	64.0 (58.0, 69.0)	57.0 (51.0, 63.0)	<0.001
Age of CRC Diagnosis (years)	81.5 (75.0, 86.0)	75.0 (69.0, 82.0)	<0.001
Body Mass Index (kg/m^2^) at cohort entry	25.8 (23.1, 28.8)	25.8 (23.1, 28.6)	0.992
BMI Class at cohort entry			
Underweight	7 (1.8%)	8 (1.5%)	0.637
Normal weight	149 (37.8%)	206 (38.9%)	
Overweight	162 (41.1%)	220 (41.5%)	
Class 1 Obesity	46 (11.7%)	70 (13.2%)	
Class 2 Obesity	20 (5.1%)	18 (3.4%)	
Class 3 Obesity	10 (2.5%)	8 (1.5%)	
Change in BMI (kg/m^2^)	−0.7 (−2.3, 0.9)	0.0 (−1.5, 1.6)	<0.001
Change in BMI (%)	−2.7 (−8.6, 3.6)	0.0 (−5.8, 6.2)	<0.001
Variance of BMI	1.5 (0.5, 3.6)	1.2 (0.4, 3.0)	0.127
Female	202 (51.3%)	294 (55.5%)	0.206
Ethnicity *			
African American	54 (13.7%)	49 (9.2%)	0.017
Hawaiian	29 (7.4%)	29 (5.5%)	
Hispanic or Latino	74 (18.8%)	94 (17.7%)	
Japanese	161 (40.9%)	212 (40.0%)	
White	76 (19.3%)	146 (27.5%)	
Diabetes	45 (11.4%)	38 (7.2%)	0.027
Smoking at Cohort Entry			
Current	62 (15.7%)	78 (14.7%)	0.308
Past	153 (38.8%)	184 (34.7%)	
Never	174 (44.2%)	260 (49.1%)	
Missing	5 (1.3%)	8 (1.5%)	
Family History of CRC	50 (12.7%)	54 (10.2%)	0.248
CRC Type			
Colon	304 (77.2%)	410 (77.4%)	0.126
Rectal	82 (20.8%)	117 (22.1%)	
Overlapping	8 (2.0%)	3 (0.6%)	
CRC Stage			
Localized	97 (24.6%)	286 (54.0%)	<0.001
Regional	136 (34.5%)	209 (39.4%)	
Distant	108 (27.4%)	23 (4.3%)	
Unknown	53 (13.5%)	12 (2.3%)	
BMI Follow-Up Duration (years)	16.0 (11.0, 17.0)	17.0 (16.0, 17.8)	<0.001
Follow-Up after CRC diagnosis (years)	1.0 (0.0, 3.0)	5.0 (2.0, 8.0)	<0.001

* Self-reported ethnic background on MEC questionnaire.

**Table 2:** Results of multivariable analysis of Cox PH.

Variable	Subset	All-Cause Mortality HR (95% CI, *p*-Value)	CA Mortality HR (95% CI, *p*-Value)
BMI Trajectory	Group 2	-	-
	Group 1	1.09 (0.82–1.44, *p* = 0.539)	0.91 (0.64–1.27, *p* = 0.566)
	Group 3	0.81 (0.61–1.08, *p* = 0.147)	0.80 (0.56–1.13, *p* = 0.200)
	Group 4	1.24 (0.90–1.71, *p* = 0.185)	1.06 (0.71–1.58, *p* = 0.760)
Sex	Female	-	-
	Male	1.09 (0.87–1.37, *p* = 0.466)	0.79 (0.60–1.06, *p* = 0.112)
Ethnicity	White	-	-
	African American	1.58 (1.09–2.29, *p* = 0.016)	1.08 (0.69–1.68, *p* = 0.749)
	Hawaiian	1.18 (0.75–1.88, *p* = 0.472)	0.48 (0.27–0.87, *p* = 0.015)
	Hispanic or Latino	1.10 (0.78–1.55, *p* = 0.577)	0.91 (0.60–1.37, *p* = 0.650)
	Japanese	1.04 (0.78–1.39, *p* = 0.781)	0.68 (0.48–0.96, *p* = 0.029)
Diabetes	No	-	-
	Yes	1.29 (0.92–1.83, *p* = 0.144)	0.79 (0.50–1.24, *p* = 0.300)
Smoking at Cohort Entry	Never	-	-
	Past	1.28 (1.01–1.63, *p* = 0.044)	1.00 (0.74–1.36, *p* = 0.979)
	Current	1.66 (1.21–2.29, *p* = 0.002)	1.33 (0.90–1.97, *p* = 0.147)
Age of CRC Dx	Mean (SD)	-	-
(Cubic Spline)	1	0.32 (0.03–3.28, *p* = 0.337)	0.31 (0.03–3.59, *p* = 0.345)
	2	7.86 (2.77–22.32, *p* < 0.001)	0.41 (0.12–1.43, *p* = 0.161)
	3	6.45 (1.25–33.41, *p* = 0.027)	3.62 (0.62–21.16, *p* = 0.151)

Results show adjusted HRs with MICE; Covariates family history of CRC, CRC type, and interaction of BMI trajectory with sex or ethnicity not included in the final multivariable model as they were not significant during model building.

**Table 3:** Results of multivariable analysis of Cox PH with competing risk.

Variable	Subset	CA Mortality HR (95% CI, *p*-Value)	Competing Risk All-Cause Mortality HR (95% CI, *p*-Value)
BMI Trajectory	Group 2	-	-
	Group 1	1.09 (0.79–1.51, *p* = 0.598)	0.99 (0.60–1.63, *p* = 0.968)
	Group 3	0.72 (0.50–1.02, *p* = 0.066)	0.96 (0.61–1.53, *p* = 0.878)
	Group 4	1.15 (0.77–1.73, *p* = 0.492)	1.45 (0.80–2.63, *p* = 0.223)
Sex	Female	-	-
	Male	0.92 (0.70–1.23, *p* = 0.583)	1.52 (0.99–2.33, *p* = 0.053)
Ethnicity	White	-	-
	African American	1.56 (1.00–2.43, *p* = 0.048)	1.53 (0.75–3.15, *p* = 0.244)
	Hawaiian	0.78 (0.45–1.36, *p* = 0.384)	2.85 (1.31–6.21, *p* = 0.009)
	Hispanic or Latino	1.06 (0.70–1.61, *p* = 0.791)	1.29 (0.69–2.42, *p* = 0.422)
	Japanese	0.90 (0.64–1.26, *p* = 0.535)	1.41 (0.84–2.36, *p* = 0.189)
Diabetes	No	-	-
	Yes	0.96 (0.58–1.57, *p* = 0.861)	1.83 (1.08–3.10, *p* = 0.026)
Smoking at Cohort Entry	Never	-	-
	Past	1.21 (0.90–1.63, *p* = 0.199)	1.34 (0.88–2.04, *p* = 0.170)
	Current	1.59 (1.10–2.32, *p* = 0.015)	1.56 (0.87–2.81, *p* = 0.138)
Age of CRC Dx	Mean (SD)	-	-
(Cubic Spline)	1	0.16 (0.01–2.25, *p* = 0.172)	11.50 (0.06–2106.96, *p* = 0.357)
	2	2.95 (0.87–10.01, *p* = 0.082)	98.04 (12.08–795.78, *p* < 0.001)
	3	2.92 (0.44–19.40, *p* = 0.265)	109.61 (3.47–3463.77, *p* = 0.008)

Results show adjusted ORs with MICE; Covariates family history of CRC, CRC type, and interaction of BMI trajectory with sex or ethnicity were not included in the final multivariable model as they were not significant during model building.

## Data Availability

The data analyzed in this study were obtained from the Multiethnic Cohort (MEC), under license for the current study. Restrictions apply to the availability of these data, and they are not publicly available. Data are, however, available from the authors upon reasonable request, with permission of the MEC.

## Supplementary Materials

The following supporting information can be downloaded at: https://www.mdpi.com/article/10.3390/cancers16172950/s1, Figure S1: Flow chart of included participants; Figure S2: Hazard of death by age of CRC diagnosis for all-cause and CRC-specific mortality; Table S1: Characteristics of persons who had cancer-specific death vs other cause mortality; Table S2: Characteristics of individuals by group.

## Abbreviations

BMI	Body Mass Index
CRC	Colorectal Cancer
GBTM	Group-Based Trajectory Modeling
HR	Hazard Ratio
IQR	Interquartile Range
MEC	Multiethnic Cohort Study
NCI	National Cancer Institute
NCDB	National Cancer Database
PH	Proportional Hazard
SEER	Surveillance, Epidemiology, and End Results
