# Supplementary material for: Investigating the Obesity Paradox in Colorectal Cancer: An Analysis of Prospectively Collected Data in a Diverse Cohort
Source: Cancers (Basel). 2024 Aug 24;16(17):2950. doi: 10.3390/cancers16172950 (PMC11394385; doi:10.3390/cancers16172950)

Supplemental Table S1. Characteristics of persons who had cancer-specific death vs other cause mortality.

Variable	*Cancer Related Death N=271*	*Non-Cancer Related Death N=123*	*P-value*
**Age (years), Median (Q1, Q3)**	63.0 (57.0, 68.0)	65.0 (60.5, 70.0)	0.001
**Age of CRC Diagnosis (years)**	81.0 (74.0, 86.0)	83.0 (76.5, 85.5)	0.247
**Body Mass Index (kg/m^2), at cohort entry**	25.7 (22.7, 28.7)	25.9 (23.6, 29.4)	0.343
**BMI Class at cohort entry**			
Underweight	4 (1.5%)	3 (2.4%)	0.846
Normal weight	107 (39.5%)	42 (34.1%)	
Overweight	110 (40.6%)	52 (42.3%)	
Class 1 Obesity	31 (11.4%)	15 (12.2%)	
Class 2 Obesity	13 (4.8%)	7 (5.7%)	
Class 3 Obesity	6 (2.2%)	4 (3.3%)	
**Change in BMI (kg/m^2)**	-0.6 (-2.2, 1.2)	-1.1 (-2.9, 0.3)	0.030
**Change in BMI (%)**	-2.5 (-8.0, 4.2)	-4.5 (-11.1, 1.2)	0.026
**Variance of BMI**	1.5 (0.4, 3.7)	1.4 (0.5, 3.6)	0.800
**Female**	147 (54.2%)	55 (44.7%)	0.083
**Ethnicity**			
African American	39 (14.4%)	15 (12.2%)	0.651
Hawaiian	18 (6.6%)	11 (8.9%)	
Hispanic or Latino	52 (19.2%)	22 (17.9%)	
Japanese	106 (39.1%)	55 (44.7%)	
White	56 (20.7%)	20 (16.3%)	
**Diabetes**	24 (8.9%)	21 (17.1%)	0.025
**Smoking at Cohort Entry**			
Current	45 (16.6%)	17 (13.8%)	0.108
Past	95 (35.1%)	58 (47.2%)	
Never	126 (46.5%)	48 (39.0%)	
Missing	5 (1.8%)	0 (0%)	
**Family History of CRC**	33 (12.2%)	17 (13.8%)	0.629
**CRC Type**			
Colon	209 (77.1%)	95 (77.2%)	1
Rectal	56 (20.7%)	26 (21.1%)	
Overlapping	6 (2.2%)	2 (1.6%)	
**CRC Stage**			
Localized	38 (14.0%)	59 (48.0%)	<0.001
Regional	92 (33.9%)	44 (35.8%)	
Distant	99 (36.5%)	9 (7.3%)	
Unknown	42 (15.5%)	11 (8.9%)	
**BMI Follow-Up Duration (years)**	16.0 (11.0, 17.0)	16.0 (11.0, 17.0)	0.140
**Follow-Up after CRC diagnosis (years)**	1.0 (0.0, 2.0)	3.0 (1.0, 6.0)	<0.001

Supplemental Table S2. Characteristics of individuals by group.

Variables	*Group 1 N=93*	*Group 2 N=152*	*Group 3 N=86*	*Group 4 N=63*	*P-value*
**Age (years), Median (Q1, Q3)**	65.0 (60.0, 70.0)	65.0 (59.0, 69.0)	65.0 (58.3, 69.0)	60.0 (57.0, 65.5)	0.027
**Age of CRC Diagnosis (years)**	82.0 (75.0, 87.0)	82.5 (75.0, 86.0)	82.5 (76.0, 85.8)	79.0 (72.5, 83.0)	0.183
**Body Mass Index (kg/m2)**	21.3 (20.2, 22.0)	25.2 (24.1, 26.0)	28.6 (27.5, 29.9)	34.6 (32.6, 37.2)	<0.001
**BMI Class**					
**Underweight**	7 (7.5%)	0 (0%)	0 (0%)	0 (0%)	<0.001
**Normal weight**	85 (91.4%)	61 (40.1%)	2 (2.3%)	1 (1.6%)	
**Overweight**	1 (1.1%)	91 (59.9%)	66 (76.7%)	4 (6.3%)	
**Class 1 Obesity**	0 (0%)	0 (0%)	17 (19.8%)	29 (46.0%)	
**Class 2 Obesity**	0 (0%)	0 (0%)	1 (1.2%)	19 (30.2%)	
**Class 3 Obesity**	0 (0%)	0 (0%)	0 (0%)	10 (15.9%)	
**Change in BMI (kg/m2)**	-0.6 (-2.0, 0.5)	-0.6 (-2.0, 0.8)	-0.9 (-2.4, 1.6)	-1.0 (-3.9, 1.9)	0.925
**Change in BMI (%)**	-3.0 (-8.6, 2.3)	-2.3 (-8.0, 3.2)	-3.2 (-8.3, 5.6)	-2.9 (-11.2, 5.3)	0.878
**Variance of BMI**	0.7 (0.2, 1.8)	1.2 (0.4, 2.5)	2.3 (1.0, 4.5)	5.0 (1.8, 10.6)	<0.001
**Female**	60 (64.5%)	60 (39.5%)	45 (52.3%)	37 (58.7%)	<0.001
**Ethnicity**					
**White**	12 (12.9%)	31 (20.4%)	17 (19.8%)	16 (25.4%)	<0.001
**African American**	7 (7.5%)	14 (9.2%)	14 (16.3%)	19 (30.2%)	
**Hawaiian**	2 (2.2%)	8 (5.3%)	9 (10.5%)	10 (15.9%)	
**Hispanic or Latino**	8 (8.6%)	28 (18.4%)	27 (31.4%)	11 (17.5%)	
**Japanese**	64 (68.8%)	71 (46.7%)	19 (22.1%)	7 (11.1%)	
**Diabetes**	4 (4.3%)	13 (8.6%)	17 (19.8%)	11 (17.5%)	0.002
**Smoking at Cohort Entry**					
**Never**	49 (52.7%)	65 (42.8%)	34 (39.5%)	26 (41.3%)	0.636
**Past**	29 (31.2%)	64 (42.1%)	35 (40.7%)	25 (39.7%)	
**Current**	15 (16.1%)	22 (14.5%)	15 (17.4%)	10 (15.9%)	
**Missing**	0 (0%)	1 (0.7%)	2 (2.3%)	2 (3.2%)	
**Family History of CRC**	16 (17.2%)	21 (13.8%)	5 (5.8%)	8 (12.7%)	0.114
**CRC Type**					
**Colon**	67 (72.0%)	115 (75.7%)	71 (82.6%)	51 (81.0%)	0.493
**Rectal**	22 (23.7%)	35 (23.0%)	14 (16.3%)	11 (17.5%)	
**Overlapping**	4 (4.3%)	2 (1.3%)	1 (1.2%)	1 (1.6%)	
**CRC Stage**					
**Localized**	22 (23.7%)	41 (27.0%)	27 (31.4%)	7 (11.1%)	0.090
**Regional**	37 (39.8%)	53 (34.9%)	21 (24.4%)	25 (39.7%)	
**Distant**	22 (23.7%)	39 (25.7%)	28 (32.6%)	19 (30.2%)	
**Unknown**	12 (12.9%)	19 (12.5%)	10 (11.6%)	12 (19.0%)	
**BMI Follow-Up Duration (years)**	16.0 (11.0, 17.0)	16.0 (11.0, 17.0)	16.0 (11.0, 17.0)	16.0 (11.0, 17.0)	0.538
**Follow-Up after CRC diagnosis (years)**	1.0 (0.0, 3.0)	1.0 (0.0, 4.0)	1.0 (0.0, 3.0)	1.0 (0.0, 2.0)	0.251

Supplemental Figure S1. Flow chart of included participants.

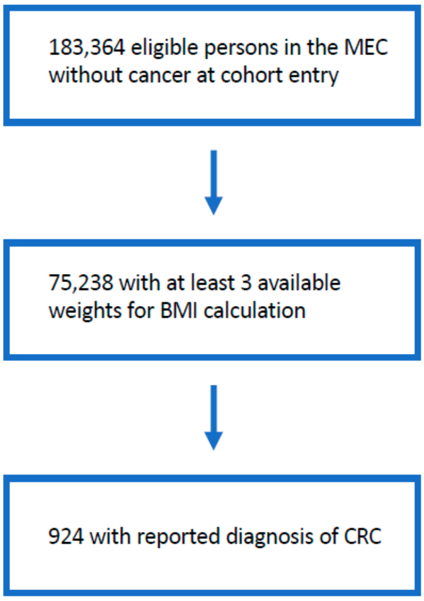

Supplemental Figure S2. Hazard of death by age of CRC diagnosis for all-cause and CRC-specific mortality.

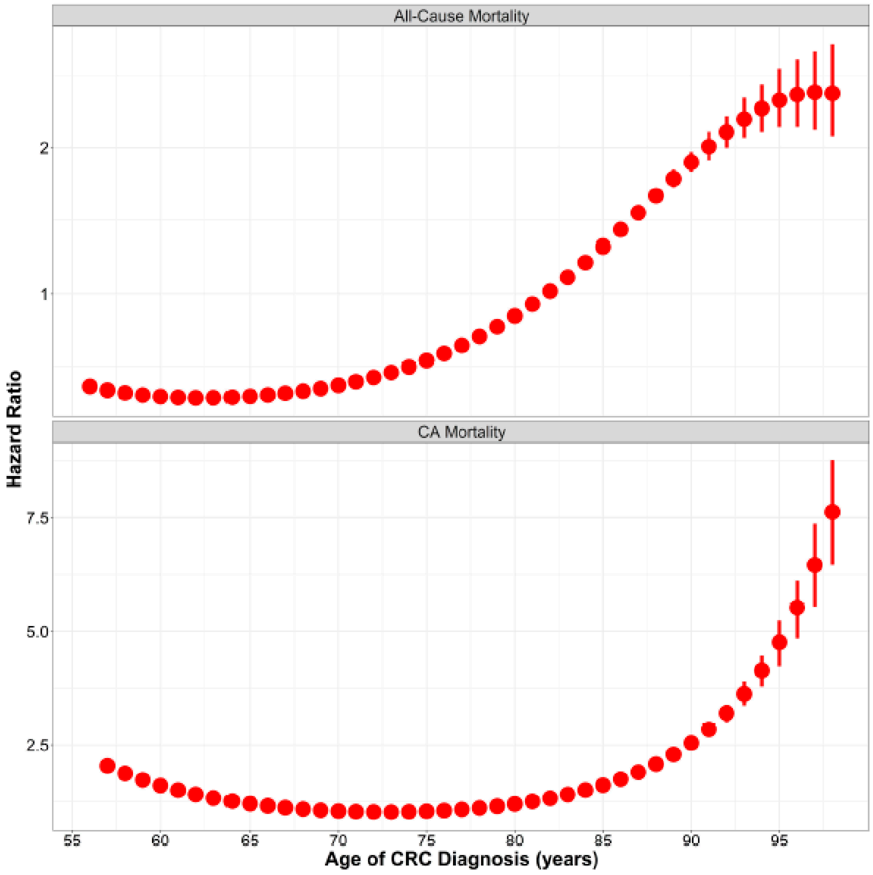

Supplement: Supplementary file 1 [file cancers-16-02950-s001.zip › cancers-3136192-supplementary.pdf]
